# Hypoxic microenvironment induced spatial transcriptome changes in pancreatic cancer

**DOI:** 10.20892/j.issn.2095-3941.2021.0158

**Published:** 2021-06-15

**Authors:** Huizhi Sun, Danfang Zhang, Chongbiao Huang, Yuhong Guo, Zhao Yang, Nan Yao, Xueyi Dong, Runfen Cheng, Nan Zhao, Jie Meng, Baocun Sun, Jihui Hao

**Affiliations:** 1 Tianjin Medical University Cancer Institute and Hospital, National Clinical Research Center for Cancer, Key Laboratory of Cancer Prevention and Therapy, Tianjin, Tianjin’s Clinical Research Center for Cancer, Tianjin 300060, China; 2 Department of Pathology, School of Basic Medical Science, Tianjin Medical University, Tianjin 300070, China

**Keywords:** Pancreatic cancer, hypoxia, spatial transcriptomic

## Abstract

**Objective:**

Hypoxia is a significant feature of solid tumors, including pancreatic ductal adenocarcinoma (PDAC). It is associated with tumor invasion, metastasis, and drug resistance. However, the spatial distribution of hypoxia-related heterogeneity in PDAC remains unclear.

**Methods:**

Spatial transcriptomics (STs), a new technique, was used to investigate the ST features of engrafted human PDAC in the ischemic hind limbs of nude mice. Transcriptomes from ST spots in the hypoxic tumor and the control were clustered using differentially-expressed genes. These data were compared to determine the spatial organization of hypoxia-induced heterogeneity in PDAC. Clinical relevance was validated using the Tumor Cancer Genome Atlas and KM-plotter databases. The CMAP website was used to identify molecules that may serve as therapeutic targets for PDAC.

**Results:**

ST showed that the tumor cell subgroups decreased to 7 subgroups in the hypoxia group, compared to 9 subgroups in the control group. Different subgroups showed positional characteristics and different gene signatures. Subgroup 6 located at the invasive front showed a higher proliferative ability under hypoxia. Subgroup 6 had active functions including cell proliferation, invasion, and response to stress. Expressions of hypoxia-related genes, *LDHA*, *TPI1*, and *ENO1*, induced changes. CMAP analysis indicated that ADZ-6482, a PI3K inhibitor, was targeted by the invasive subgroup in hypoxic tumors.

**Conclusions:**

This study is the first to describe hypoxic microenvironment-induced spatial transcriptome changes in PDAC, and to identify potential treatment targets for PDAC. These data will provide the basis for further investigations of the prognoses and treatments of hypoxic tumors.

## Introduction

Pancreatic ductal adenocarcinomas (PDACs) are leading causes of cancer death with a 5-year survival of < 10%^1,2^. Approximately 10%–15% of newly diagnosed patients are affected^1,3,4^. There is no efficient treatment to improve the prognoses of PDACs; hence, the majority of PDAC patients will eventually die from the disease^5–7^. Genomic and transcriptomic studies have revealed that critical gene mutations or aberrant signaling pathways drive PDAC, such as KRAS driver mutations and frequent inactivation of tumor suppressors, including TP53, SMAD4, and CDKN2A^8–11^. Other rare mutations have also been identified in unbiased analyses of PDACs. These diverse gene mutations converge on specific pathways and processes, including TGF-β, Wnt, Notch, ROBO/SLIT signaling, and chromatin remodeling and DNA repair pathways^12,13^. Inactivating mutations of chromatin modifiers have been identified in PDACs. These modifiers include histone modification enzymes and SWI/SNF-mediated chromatin remodeling complexes^14^. Unfortunately, none of them have been used as clinical targets, mainly due to limited understanding of their potential roles in PDAC progression^1,15^.

Hypoxia plays a key role in regulating the tumor microenvironment by defining the behaviors of many solid malignant tumors^16^. It is also associated with invasion, metastasis, poor clinical prognosis, and resistance to therapies in many malignant tumors^17^. The hypoxic microenvironment induces the expression of gene products involved in angiogenesis, metabolism, invasion, and metastasis in PDACs. PDAC cell lines grow well in hypoxic culture conditions (0.1% O_2_), and mitochondria adapt to this situation using respiratory chain supercomplexes^18^. Thus, identifying hypoxia-regulated genes and the mechanisms involved are important both for understanding cancer evolution and for improving the prognosis or development of hypoxia-sensitive prodrugs for the treatment of PDAC patients^19^.

Traditional transcriptomics using RNA sequencing result in an averaged transcriptome and the loss of spatial information. Single cell genomics is a powerful tool to characterize genetic and functional heterogeneities, for reconstruction of evolutionary lineages, and to detect rare subpopulations^20^. The scRNA-seq studies in tumors have provided new insights into tumor heterogeneity and the existence of different subpopulations, which are pivotal concepts for identifying detailed tumor-related mechanisms. However, it does not reflect the spatial distribution of tumor tissues. The positional context of gene expression is of key importance for understanding tumor hypoxia and hypoxia-induced gene expressions. Stahl et al.^21^ introduced the spatial transcriptomics (STs) method, which allows for quantification of the mRNA population in the spatial context of intact tissues^22^.

In this study, we established human PDAC engrafts in mice ischemic hind limbs and used ST tissue to investigate ST changes in PDAC in a hypoxic microenvironment. Together, these results identified hypoxia-induced spatial heterogeneity and its related clinical significance in PDAC. This study will provide the basis for further investigations of molecular tumor signatures for the prognosis and treatment of PDAC patients.

## Materials and methods

### Cells and tumors engrafted in the ischemic hind limbs of nude mice

Animal experiments were approved by the ethics committee of Tianjin Medical University (Approval No. 8207110937). All steps were carefully administered to protect the welfare of the animals and to minimize suffering. Details are provided in the Supplementary information.

### Spatial transcriptomics

#### Slide preparation

ST slides were printed with four capture areas (6.5 × 6.5 mm), each with 4,999 capture spots of barcoded primers (10× Genomics, Pleasanton, CA, USA). The spots had a diameter of 100 µm and were arranged in a centered rectangular lattice pattern. Each spot contained millions of oligonucleotides with the following features: a 30 nucleotide poly(dT) sequence for the capture of polyadenylated mRNA molecules, a 12 nucleotide unique molecular identifier (UMI) for the identification of duplicate molecules that arose during the library preparation and sequencing process; a 16 nucleotide spatial barcode that was shared by all oligonucleotides within each individual gene expression spot; and a partial TruSeq Read 1 sequence, for use during the library preparation and sequencing steps of the workflow.

#### Tissue preparation

The ST protocol was optimized for Panc-1 engrafted tissue according to 10× Genomics. Briefly, tumors were harvested, cut into 5 mm-thickness tissue blocks and immediately frozen on dry ice. Tumor blocks embedded with optimal cutting temperature reagent were cryosectioned at a thickness of 10 µm and attached to the capture areas before proceeding to the next step.

#### Fixation, staining, and imaging

Sectioned slides were incubated at 37 °C for 1 min and fixed in methanol for 10 min at −20 °C. For staining, the sections were incubated in isopropanol (MilliporeSigma, Burlington, MA, USA) for 6 min, Mayer’s hematoxylin (Agilent, Santa Clara, CA, USA) for 7 min, Bluing Buffer (Dako) for 1 min, and eosin (Sigma-Aldrich, St. Louis, MO, USA) diluted 1:5 in Tris-base (0.45 M Tris, 0.5 M acetic acid, pH 6.0) for 1 min. The slides were washed with deionized water after each of the staining steps. After air-drying, the slides were mounted with 85% glycerol and coverslips. Hematoxylin and eosin-stained images were recorded at 40× magnification using a digital slice scanner (Hamamatsu, San Jose, CA, USA). The coverslip was removed after imaging by immersing the slides in RNase-and DNase-free water.

#### Tissue permeabilization

The slides were inserted into slide cassettes to separate the tissue sections into individual reaction chambers (wells). For pre-permeabilization, the sections were incubated at 37 °C for 18 min with 70 µL permeabilization enzyme. The wells were washed with 0.1 × saline sodium citrate (SSC) (Sigma-Aldrich).

#### Reverse transcription, spatial library preparation, and sequencing

SSC was removed, and 75 µL reverse transcription Master Mix (Sigma-Aldrich) was added to each well. Reverse transcription was conducted according to the manufacturer’s protocol. After reverse transcription, the wells were washed with 0.1× SSC. Sections were then incubated in 75 µL 0.08 M KOH for 5 min at room temperature, and then were incubated in 75 µL Second Strand Mix (Thermo Fisher Scientific, Waltham, MA, USA) for 15 min at 65 °C. After removal of the Second Strand Mix, 100 µL Buffer EB were added, and sections were placed in 35 µL 0.08 M KOH for 10 min at room temperature. The samples were then transferred from each well to a corresponding tube containing Tris-HCl (1 M, pH 7.0). Next, 1 µL of the sample was added to the qPCR plate well containing the KAPA SYBR FAST qPCR Master Mix (KAPA Biosystems, Cape Town, South Africa). The qPCR was performed following the manufacturer’s protocol, and the optimal number of cycles was determined. Next, 65 µL of the cDNA Amplification Mix (Takara Bio, Mountain View, CA, USA) was added to the remaining sample, which was then incubated for 12 cycles according to the recommended protocol.

#### Library preparation and RNA sequencing

After the identities of the cDNA amplification products were confirmed, the sequencing library was constructed using a Library Construction KIT (10× Genomics). First, the cDNA was chemically knocked-out. The cDNA fragment was then cut into 200~300 bp fragments, the cDNA fragments were segmented, and their terminals were repaired and added. The cDNA fragments were then screened. The P7 adapter was connected and introduced into the sample index using PCR amplification. Finally, a sequence library was obtained. Sequencing was performed on an Illumina Hiseq 3000/4000 (Illumina, San Diego, CA, USA) with a 150 bp pair-end run by Quick Biology (Pasadena, CA, USA). A data quality check was performed using the SAV (Illumina). Demultiplexing was performed using the Bcl2fastq2 v 2.17 program (Illumina).

### RNA sequencing analysis

In this study, the official Space Ranger software (10× Genomics) was used for data preprocessing, quantitative gene expression analysis, and point identification. Sequencing data preprocessing included filtering the sequenced product, evaluating the quality of sequencing data, and calculating the sequence length distributions. The web-based ST spot detector software, Space Ranger (10× Genomics), was used to identify the spatial barcode markers in Reads1 and UMI markers of different transcripts. Read2 was aligned to the reference genome, human GRCh38 v86, and mouse mm10, using the transcriptome-specific STAR alignment software (starsoftware.co). The sequence with a unique alignment position was selected for subsequent analysis. Space Ranger (10× Genomics) produced the bright field slide image of a single capture area and the fastq sequence, distinguished by tissue, background, and the detected spot barcodes. The gene spot matrix was generated by using Viscum spatial barcodes, and then point clustering and gene expression analysis were performed.

Seurat software (https://cran.r-project.org/web/packages/Seurat/index.html) was used to analyze and cluster the four samples. Low quality data were then filtered. Principal Component Analysis (PCA), including the t-Distributed Stochastic Neighbor Embedding (t-SNE) and Uniform Manifold Approximation and Projection (UMAP) algorithms, were used to reduce and visualize the dimensions of the data. All spots from the four samples were clustered according to differentially-expressed genes, which were expressed in over 25% of the spots with LOGFC values greater than 0.25. Heat maps were generated with Seurat software using default hierarchical clustering of read counts.

### Identification of cluster-specific hypoxia genes and marker genes

The hypoxia gene was curated from the literature (**Supplementary Table S2**). Gene sets with false discovery rate-adjusted *P*-values < 0.05 were considered significantly enriched in the related clusters. Kyoto Encyclopedia and Genes and Genomes (KEGG) and Gene Ontology (GO) slim were used to analyze the signaling pathway information. Data from 1,222 cases of The Cancer Genome Atlas (TCGA) breast cancer were downloaded. The top 100 cases with the highest expressions and 100 cases with the lowest expressions of TCGA were selected for Gene Set Enrichment Analysis (4.0.3)^23^. Cluster-specific hypoxia genes were then analyzed (ftp.broadinstitute.org://pub/gsea/gene_sets/h.all.v7.1.symbols.gmt).

Marker genes were identified based on a comprehensive analysis of the database and gene ranks of LOGFC values in differentially-expressed genes of the clusters. The top 200 differentially expressed genes and cluster-specific hypoxia genes of each cluster were submitted to the STRING website (https://string-db.org/), and the human protein interaction network provided by the STRING database was imported into Cytoscape 3.8.0 (https://cytoscape.org/release_notes_3_8_0.html) software. The MCODE app (http://apps.cytoscape.org/apps/mcode) was used to score and enrich the dense region groups of the protein interaction network. The BOTTLENECK algorithm of the cytoHubba app (http://apps.cytoscape.org/apps/cytohubba) was used to identify the top 20 molecules in the protein interaction network. Ten genes with higher rank, MCODE score, and cytoHubba score were identified as marker genes for each cluster. Pearson’s correlation was used to identify the relationship between cluster-specific hypoxia and marker genes. Correlation heat maps were provided by the HIPLOT website (https://hiplot.com.cn/basic/cor-heatmap).

### Clinical significance of cluster-specific hypoxia genes and marker genes in human PDAC

The prognostic values of cluster-specific hypoxia and marker genes were evaluated using the KM-Plotter database^24^. Approximately 178 PDAC patient samples were divided into 2 groups according to the median value of marker gene expressions from the gene chip. The 2 patient cohorts were compared using a Kaplan-Meier survival plot, and the hazard ratios with 95% confidence intervals and log-rank *P*-values were calculated.

### Connectivity map (cMAP) query

To identify candidate compounds for hypoxic tumor treatment, we used an online cMap analysis (https://clue.io/query)^25^. The marker genes of all subgroups in the hypoxia and control groups were submitted and queried against the L1000 beta dataset released on December 17, 2020. The expression signatures of 9 human cancer lines treated with 2,837 chemical drugs were compared to the genes that were submitted and scored. CMap drug-gene expression profiles with negative mean scores reversed (or opposed) gene expression profiles were compared with the submitted data.

## Results

### The STs of human Panc-1 engrafts

**Figure 1:**
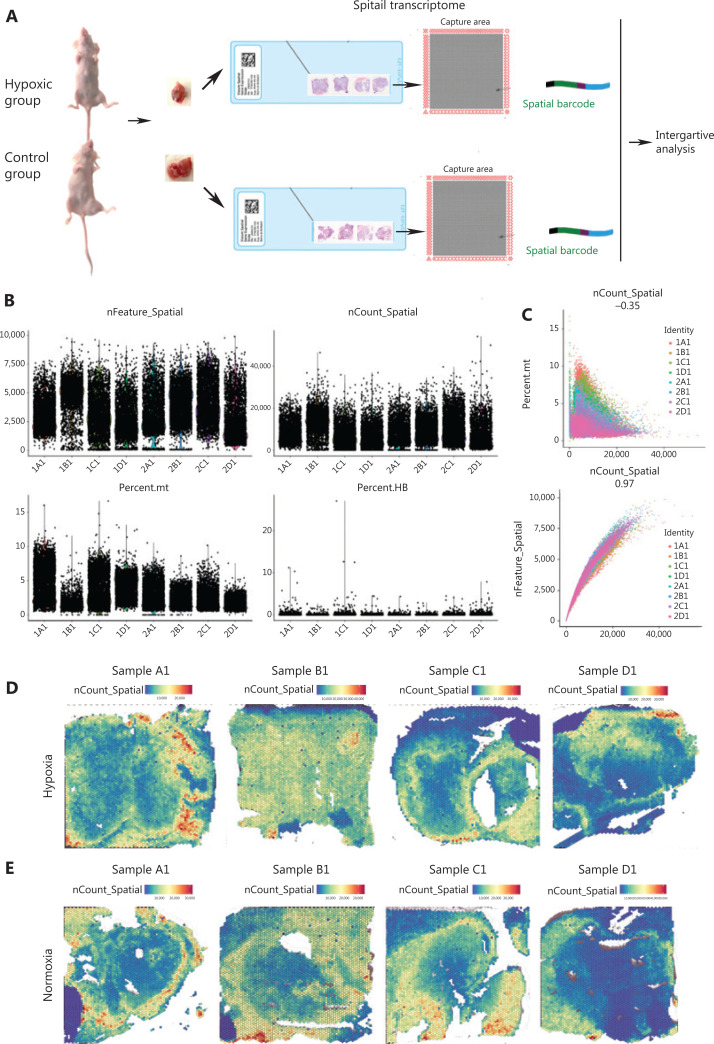
A spatial transcriptomic atlas of human pancreatic ductal adenocarcinoma (PDAC) Panc-1 engrafts. (A) Workflow of PDAC sample processing for spatial transcriptomics. (B) Distribution of all expression gene numbers in 8 samples, distribution of all expression genes in 8 samples, distribution of mitochondrial genes in 8 samples, and distribution of hemoglobin gene expressions in 8 samples. Samples marker in the hypoxia group: 1A, 1B, 1C and 1D; Samples marker in the control group: 2A, 2B, 2C and 2D. (C) Scatter plot of the correlations between gene expressions and the mitochondrial gene expression ratios and gene numbers. (D) Spatial distributions of the numbers of expressed genes in 4 samples of the hypoxia group. (E) Spatial distribution of the numbers of expressed genes in 4 samples of the control group.

To assess the spatial organization of PDAC tumor cell populations under different hypoxic conditions, we performed ST on 8 sections from PDAC engraftments in ischemic hind limbs and controls. Transcriptomes from 15,731 spots across four sections in the hypoxia group and 14,951 spots in the control group were obtained, and the STAR group-specific alignment software was matched to the Read2 in the reference genomes, human Gsh38 and mouse mm10. Sequences with unique alignment positions were selected for subsequent analyses. The data were obtained at a median depth of 2,178.8 human genes/spot and 730.8 mouse genes/spots in the hypoxia group, and 2,541.5 human genes/spot and 730.5 mouse genes/spot in the control group. Seurat software was used to analyze the ST data using data dimensionality reduction including PCA, t-SNE, and UMAP. **Figure 1B** shows the gene number distribution, expression distribution, and mitochondrial and hemoglobin gene expression ratios of all spots. The spatial gene number distributions and their respective expression distributions have been shown in 8 sections (**Figure 1C to 1F**).

**Figure 2:**
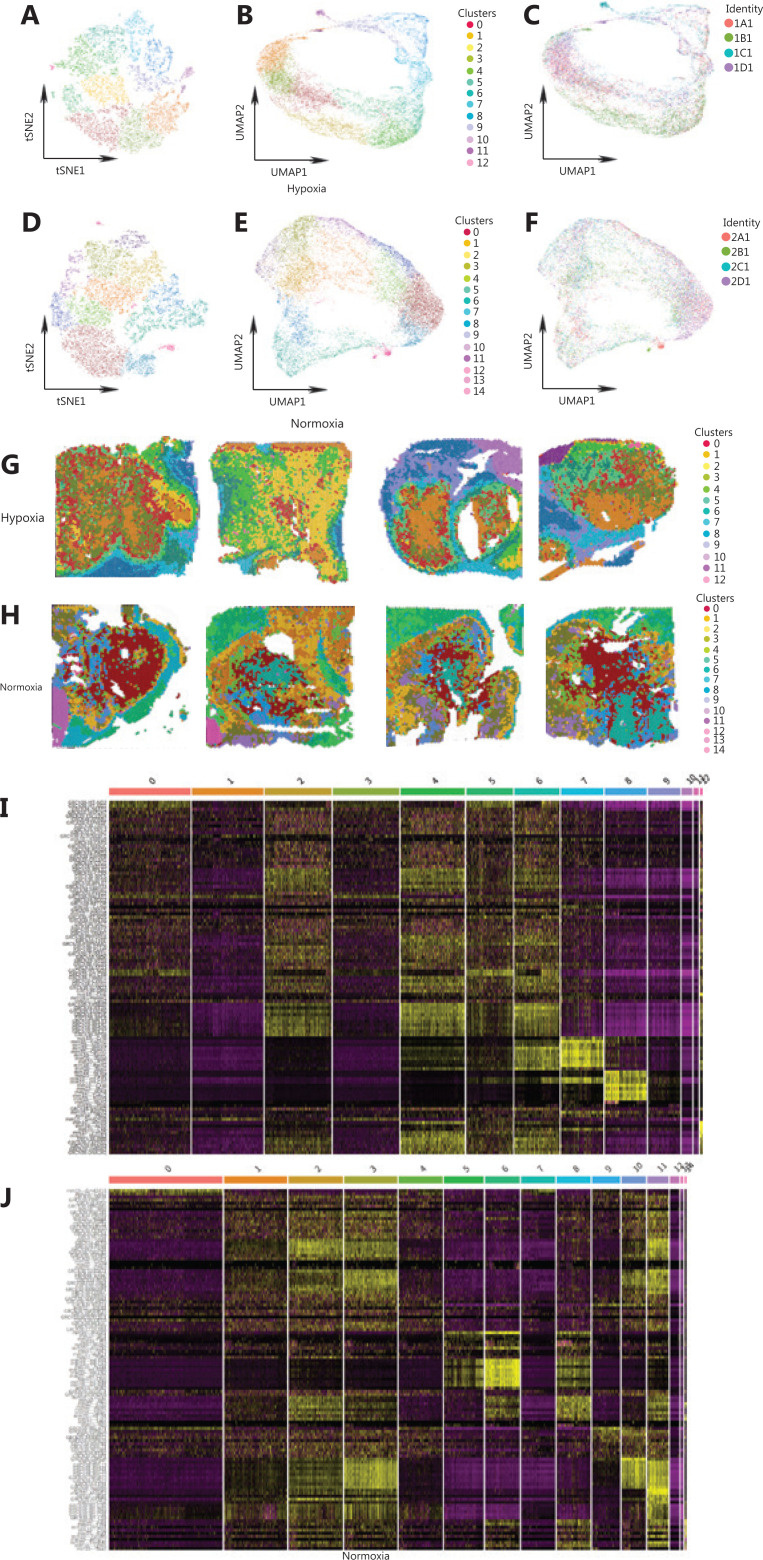
Classification of Panc-1 engrafts. (A) The t-NSE clustering graph of the hypoxia group. (B) Uniform Manifold Approximation and Projection (UMAP) clustering graph of the hypoxia group. (C) UMAP clustering graph of every sample in the hypoxia group. (D) The t-NSE clustering graph of 4 samples of the hypoxia group. (E) UMAP clustering graph of the control group. (F) UMAP clustering graph of the control. (G) UMAP clustering graph of every sample in the control group. (H) UMAP clustering graph of 4 samples of the control group. (I) Spatial distribution of all clusters in 4 samples of the hypoxia group. (J) Spatial distribution of all clusters in 4 samples of the control group.

According to the results of t-SNE and UMAP, the spots of 4 sections in the hypoxia group were grouped into 13 clusters, and the spots of 4 sections in the control were grouped into 15 clusters (**Figures 2A to 2F**). **Figures 2G and 2H** indicate the distribution of clusters under different hypoxia conditions. **Figures 2I and 2J** show the top 10 differentially expressed genes in the hypoxia and the control groups.

### The changes of heterogeneities of STs of PDACs in different hypoxic microenvironments

**Figure 3:**
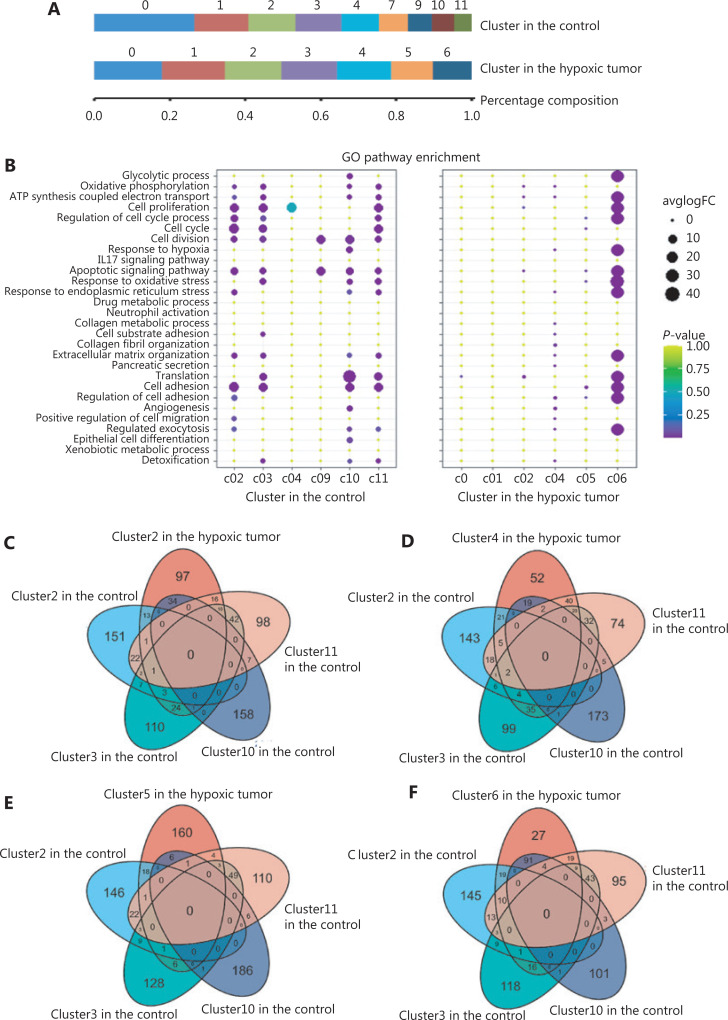
Comparison of marker genes and selected pathways of tumor-dependent clusters of the hypoxia and control groups. (A) The composition and proportion of tumor subgroups of the hypoxia and control groups. (B) Dot plot of the top 4 Gene Ontology terms of differentially-expressed genes in tumor-dependent clusters of the hypoxia and control groups. (C) A Venn diagram of marker genes in cluster 2 of the hypoxia group and clusters 2, 3, 10, and 11 of the control group. (D) A Venn diagram of marker genes in cluster 4 of the hypoxia group and clusters 2, 3, 10, and 11 of the control group. (E) A Venn diagram of marker genes in cluster 5 of the hypoxia group and clusters 2, 3, 10, and 11 of the control group. (F) A Venn diagram of marker genes in cluster 6 of the hypoxia group and clusters 2, 3, 10, and 11 of the control group.

To investigate the changes in ST heterogeneity of PDACs induced by hypoxic microenvironments, clusters in the 2 groups were analyzed. Tumor cells in the ischemic hind limb were enriched in 7 subgroups, compared to 9 subgroups in the control group (**Figure 3A**). The number of tumor cell subgroups decreased in the hypoxia group. Furthermore, GO enrichment analysis was used to compare the characteristics of different pancreatic cancer cell subgroups in the hypoxia and control groups.

In the control group, the cell functions of each subgroup were diverse. The hypoxic microenvironment induced the concentration of cell subgroups in a few subgroups (**Figure 3B**). Because proliferation was found to be the main feature of PDAC, the relative signaling pathways were validated in this study. It was found that the proliferative activities of multiple cell subsets in the control group were stronger than those in the hypoxia group (**Figure 3B**). The unique functions of subgroups 2,3, 4, and 11 in the control were related to cell cycle and cell proliferation, and the proliferative abilities of the cell subsets in the hypoxia group were related to subgroup 6 (**Figure 3B**). Specifically, genes for subgroup 10 in the control group were enriched for GO terms such as response to hypoxia, angiogenesis, and cell adhesion, emphasizing their potential functions in migration and metastasis (**Figure 3B**). The functions of the genes for subgroup 9 in the control group were associated with cell division and apoptosis (**Figure 3B**). Notably, GO function analysis indicated that only subgroup 6 had active functions. Genes for subgroup 6 in the hypoxia group were enriched for cell proliferation, invasion, and the response to stress (**Figure 3B**). We also found that the genes expressed in subgroup 4 of the hypoxia group were related to angiogenesis, extracellular matrix organization, and collagen organization, indicating a possible association with the immune response (**Figure 3B**).

Based on the differences in tumor heterogeneities between the 2 groups, we compared differentially-expressed genes in the subpopulations of these groups. The results showed that gene expression of subgroup 5 in the hypoxia group was the least coincident with other groups (**Figures 3C to 3F**). This indicated that pancreatic cancer in a hypoxic microenvironment produced a new functional subgroup.

### The difference of hypoxia gene expression patterns in the hypoxia and control groups

Because pancreatic cancer cells have a strong tolerance to hypoxic stress, we investigated hypoxia-related gene signatures in different groups; 35 hypoxia-related genes reported in the literature were selected and analyzed for their expression and spatial distributions. There were 28 genes expressed in this PDAC model, and the expression of 7 genes was considered to be absent. Notably, these genes were differentially-expressed in these clusters (**Supplementary Figure S1**). The distributions of hypoxia-related genes among the subgroups were uneven. Hypoxia-related genes such as *ENO1*, *LDHA*, *TPI1*, *ALDOA*, *MIF*, and *PGK1* were highly expressed in subgroup 6 of the hypoxia group and in subgroup 10 of the control group (**Supplementary Figure S1**).

**Figure 4:**
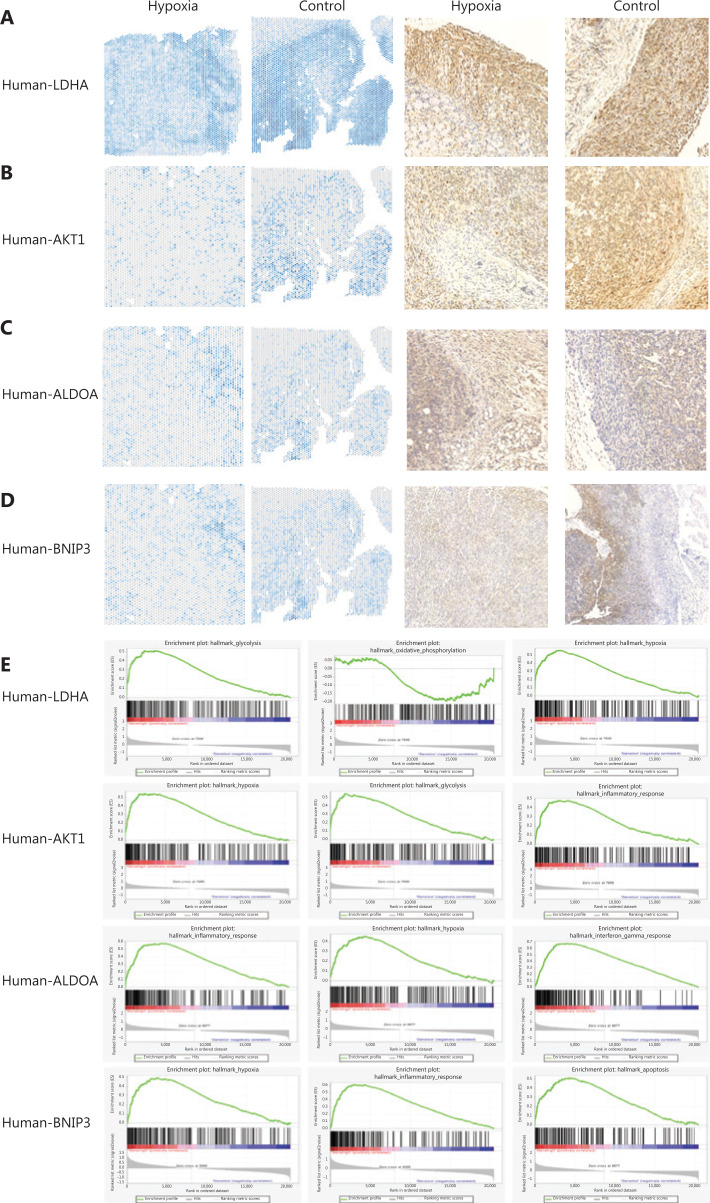
The validation of *LDHA, AKT, ALDOA*, and *BINP3* expressions in pancreatic ductal adenocarcinomas (PDACs). (A) Spatial distribution of *LDHA* in the hypoxia and control groups. Immunohistochemistry (IHC) for human *LDHA* in the hypoxia and control groups. (B) Spatial distribution of *AKT* in the hypoxia and control groups. IHC for human *AKT* in the hypoxia and control groups. (C) Spatial distribution of *ALDOA* in the hypoxia and control groups. IHC for human *ALDOA* in the hypoxia and control groups. (D) Spatial distribution of *BINP3* in the hypoxia and control groups. IHC for human *BINP3* in the hypoxia and control groups. (E) The GSEA of *LDHA, AKT1, ALDOA*, and *BINP3* in human PDAC.

The genes significantly associated with hypoxia were validated by immunohistochemistry (IHC) staining. The results in both ST and IHC analyses revealed that *LDHA* expression in the tumor boundary in both groups was higher than that in the tumor center (**Figure 4A**). *AKT* expression in the hypoxia group was upregulated compared to that in the control group (**Figure 4B**). In contrast to *AKT*, *ALDOA* expression in the hypoxia group was lower than that in the control group (**Figure 4C**). There was no significant difference in *BINP3* expression, which is involved in cell death-related signaling pathways, in either group (**Figure 4D**).

To determine the signaling pathways of genes significantly associated with hypoxia, GSEA analyses were performed in TCGA PDACs. Although the hypoxia status was different in different clusters, LDHA, AKT, ALDOA, and BINP3 participated in hypoxia, glycolysis, the inflammatory response, and oxidative phosphorylation signaling pathways, respectively, in human PDACs (**Figure 4E**).

### Spatial distribution and cross-talk of tumor gene expressions in different hypoxic microenvironments in PDACs

To investigate the differences in gene expressions in different areas between the hypoxia and control groups, the marker genes were enriched in each subgroup in both groups. GO slim and KEGG signaling pathway analyses were used to analyze the top 100 genes in each subgroup, showing that tumor cells in all subgroups in the hypoxia group had a high metabolism (**Supplementary Figure S2**).

To determine how hypoxia and tumor metabolism affected the gene expressions and gene network operations in different subgroups, we used correlation analysis, String map, Mcode, and cytoHubba in the Cytoscape software to analyze the relationships between the top 100 genes and the differentially-expressed hypoxia genes in every subgroup. **Supplementary Figures S3 to S6** show the gene cross-talk networks and the genes with the highest cytoHubba score (bottleneck algorithm) in different subgroups of the hypoxic tumors.

Based on the differences in tumor heterogeneities between the 2 groups, we compared differentially-expressed genes in subpopulations of the 2 groups. Differentially expressed genes in subgroup 4 of the hypoxia group were related to ATP synthesis-coupled electron transport, oxidative phosphorylation, the response to endoplasmic reticulum stress, and the response to oxidative stress (**Supplementary Figure S4**). *SOD1*, the key marker gene in this subtype, was related to the expression of several heat shock proteins, such as *HSP90AA* and *HSPD1* (**Supplementary Figure S4**). The marker genes of subgroup 5 in the hypoxia group were involved in protein synthesis, protein extracellular transport, and gland secretion (**Supplementary Figure S5**). They are responsible for normal pancreatic ductal cell function. The marker gene *TOP2A* in subgroup 5 was correlated with *EGFR*, *ALDH18A*, and *FBL* (**Supplementary Figure S5**). Marker genes of subgroup 6 in the hypoxia group were enriched for cell proliferation, invasion, and the response to stress. There were interactions between the marker genes, *GAPDH*, *EEF1A1*, *SERPINE1*, and hypoxia-related *TPI1* (**Supplementary Figure S6**). There were positive associations between *LDHA*, *TPI1*, and *ENO1* expressions in all the subgroups, which were involved in signaling pathways regulating glycolysis, glycolytic processes, pyruvate metabolism, ADP metabolism, and NADH regeneration.

### Clinical relevance of marker gene expressions of different clusters in PDACs

**Figure 5:**
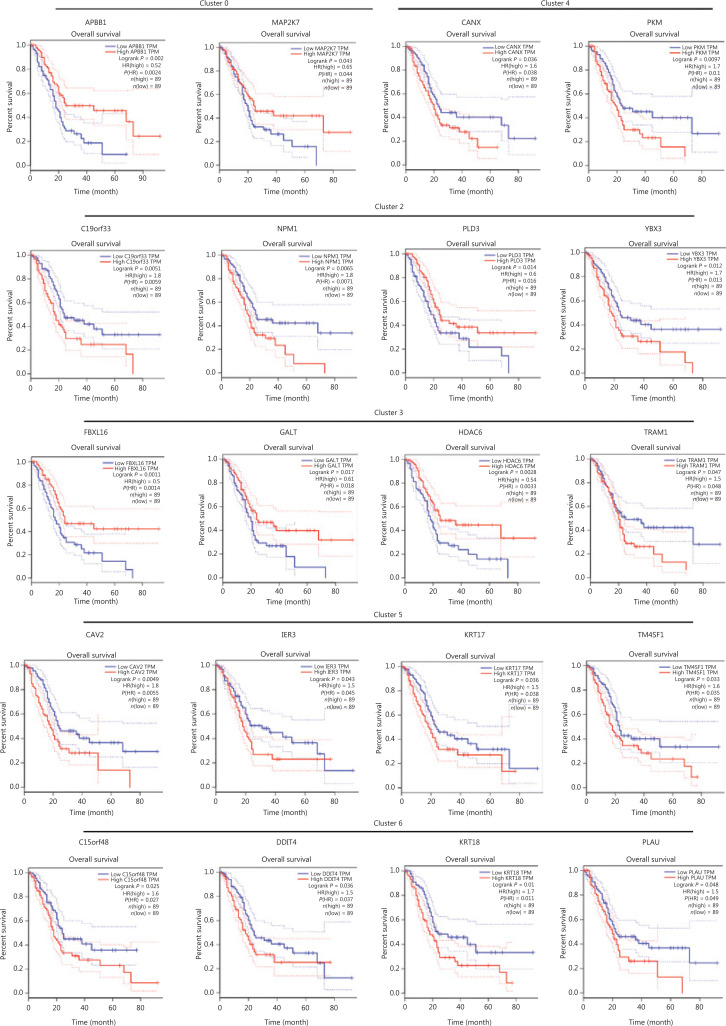
Kaplan-Meier survival plot of marker genes of clusters 0, 2, 3, 4, 5, and 6 of the hypoxia group in human pancreatic ductal adenocarcinomas (PDACs).

The KM-plotter website was used to analyze and evaluate the clinical significance of marker genes of subgroups in the hypoxia group. According to 178 PDACs in the KM-plotter database, low expression of *APBB* and *MAP2K7* in subgroup C0 of the hypoxia group was associated with poor prognoses for PDAC patients (**Figure 5**). High levels of *C19orf33*, *YBX3*, and *NPM1* in subgroup 2 of the hypoxia group indicated poor prognoses for PDAC patients (**Figure 5**). Downregulation of *PLD3* was associated with poor prognoses for PDAC patients (**Figure 5**). High expression of *GALT*, *FBXL16*, and *HDAC6* from subgroup 3 in the hypoxia group was associated with good prognoses for PDAC patients, while upregulation of *TRAM1* indicated poor prognoses for PDAC patients (**Figure 5**). Upregulated *CNAX* and *PKM* expression in subgroup 4 in the hypoxia group was associated with poor prognoses for PDAC patients (**Figure 5**). High expression of *TM4SF1*, *CAV2*, *IER3*, and *KRT17* in subgroup C5 in the hypoxia group indicated poor prognoses for PDAC patients (**Figure 5**). High levels of *C15orf48*, *PLAU*, *DDIT4*, and *KRT18* in subgroup 6 in the hypoxia group indicated poor prognoses for PDAC patients (**Figure 5**).

### Identification of potential drugs for hypoxia-induced PDACs

**Figure 6:**
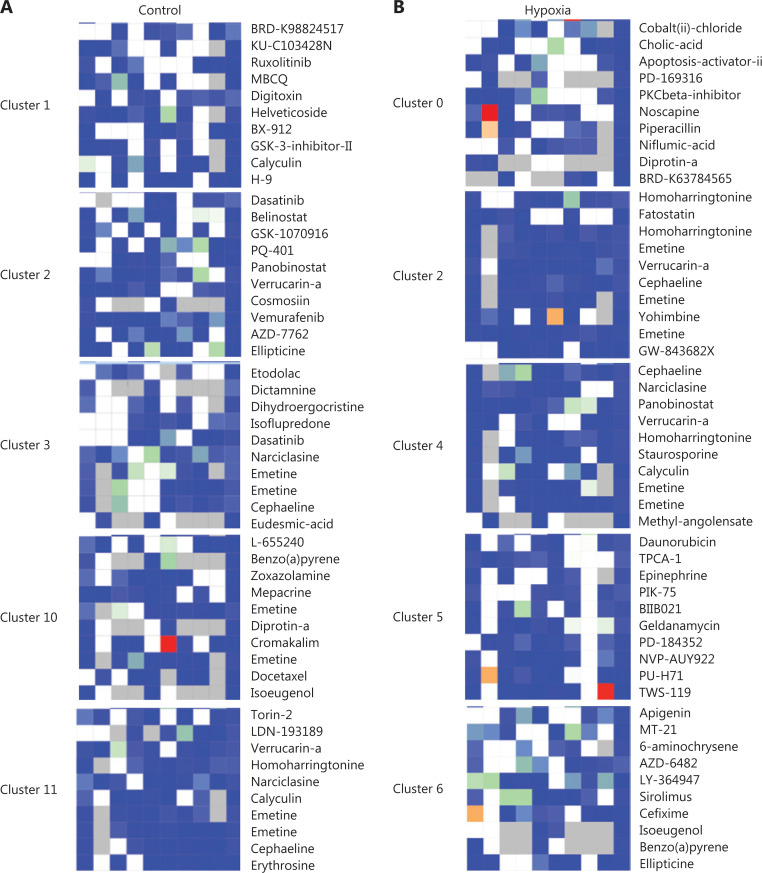
Comparison of drug candidates of tumor-dependent clusters of the hypoxia and control groups. (A) Drug candidates of tumor-dependent clusters of the control group. (B) Drug candidates of tumor-dependent clusters of the hypoxia group.

Although the growth of transplanted tumors is limited by ischemia and hypoxia, we used the online Connectivity Map (CMap) software to search for the original drug involved in hypoxia-induced PDAC in cases of tumor relapse. Query CMap is a chemical genomics database that collects gene expression profiles from cultured human cells treated with small molecules^25,26^. A CMap analysis was performed in which we searched for drugs that had a gene expression pattern negatively correlated to the hypoxia-induced pancreatic cancer (PNCA). The top 10 potential drugs for each subgroup in the hypoxia and the control groups are shown in **Figure 6**.

## Discussion

PDAC is characterized by a high degree of intratumoral heterogeneity, which constitutes the main obstacle for effective PDAC treatment^27^. Thus, it is highly desirable for intratumoral heterogeneity and the underlying mechanisms that are pivotal for PDAC prognostic improvement. In this study, we established an ischemic comprehensive gene expression atlas of various areas of hypoxia-induced PDAC, and characterized the features of spatial gene expression profiles in each subgroup based on ST and scRNA-seq analyses.

Hypoxia has a significant impact on tumor heterogeneity^28,29^. In this study, we found that the growth of pancreatic cancer xenografts was significantly inhibited in the ischemic hind limb model, but the tumor cells still survived. This was related to the severe fibrosis, fewer blood vessels, and strong hypoxia tolerance of pancreatic cancer cells^30,31^. Therefore, this study compared the transcriptomes of the hypoxia and control groups to characterize the effect of hypoxia on transcriptome heterogeneity of pancreatic cancer. The results indicated that tumor cell subgroups decreased in the hypoxia group. Furthermore, GO enrichment analysis showed that in the control group, the cell function of each subgroup showed diversity. Tumor cells in a hypoxic microenvironment undergo clonal selection^28^, resulting in the reduction of cell subgroups and functional simplification. The prominent proliferative activity of tumor cells is an important feature of pancreatic cancer. In a normoxic environment, many subpopulations of pancreatic cancer cells express genes of the cell cycle and cell proliferation pathways, and have strong proliferative activities. Except for the proliferation-associated signaling pathway, the genes for subgroup 10 in the control group were enriched in response to hypoxia, angiogenesis, and cell adhesion, emphasizing their potential functions in migration and metastasis. The functions of genes in subgroup 9 in the control group were responsible for cell division and apoptosis. Only subgroup 6, located at the invasive front, showed proliferative ability under hypoxia.

Accumulating evidence has highlighted the influence of the microenvironment on cancer progression and evolution^32^. Glioblastoma cells in hypoxic regions of tumors overexpress the epidermal growth factor receptor, and the vascular regions express the platelet-derived growth factor receptor α^33^. Similarly, hypoxia induced by anti-angiogenic agents, such as sunitinib and bevacizumab, can increase the population of cancer stem cells in breast cancers^34^. In the present study, the hypoxic microenvironment induced heterogeneous changes and new functional subgroups in pancreatic cancer^35–37^. The gene expression of subgroup 5 in the hypoxia group was the least coincident with the other groups. The signaling pathways of the new functional subgroups in the hypoxia group were associated with protein synthesis, extracellular protein transport, and gland secretion. These genes are related to normal pancreatic functions, supporting the possibility that the new functional subgroup possesses a certain degree of the differentiation and maturation of the pancreatic epithelium. Tumor cells of subgroup 4 in the hypoxia group enhanced their abilities to resist endoplasmic reticulum stress and oxidative stress by activating ATP synthesis-coupled electron transport and oxidative phosphorylation. The key marker gene, *SOD1*, in this subtype was related to the expression of several heat shock proteins, such as *HSP90AA* and *HSPD1* (**Supplementary Figure S4**). Notably, subgroup 6 at the invasive front of the hypoxic tumor had more active functions regulating cell proliferation, invasion, and the response to stress. The morphology of PNCA in the hypoxia group also showed that subgroup 6 was located at the junction of tumor tissue and the muscle, which was the most invasive part. These results indicated that pancreatic cancer cells in subgroup 6 had a stronger ability for survival and invasion. The new functional subpopulations and aggressive subpopulations may be responsible for the survival, proliferation, and invasion of PCNA under hypoxic stress.

Pancreatic cancer is a type of tumor characterized by extreme hypoxia^38^. The Wagner effect is an important feature of pancreatic cancer metabolism. We analyzed the spatial distribution of 35 hypoxia-related genes between the hypoxia and the control groups^39–41^, and found that the distribution of hypoxia-related genes among the subgroups was uneven. Hypoxia-related genes such as *ENO1*, *LDHA*, *TPI1*, *ALDOA*, *MIF*, and *PGK1* were highly expressed in subgroup 6, the most invasive subgroup of the hypoxia group and subgroup 10 of the control group. *LDHA* was one of the most highly expressed genes, and other genes are also important for the regulatory mechanisms in glycolysis^42^. The other upregulated genes were key enzymes^43,44^. GO analysis also showed that glycolysis, pyruvate metabolism, NADH regeneration, and other metabolic pathways were significantly activated in hypoxia group 6, suggesting that hypoxia-induced glycolysis was an important metabolic characteristic of pancreatic cancer cells, which met the biological functional requirements of these cells. In the process of tumor proliferation, invasion, and metastasis, considerable synthesis is needed, such as amino acid and nucleotide synthesis^45,46^. In addition to consuming a large amount of ATP, carbon and NADPH equivalents are also needed as raw materials^47,48^. Glycolysis can not only produce ATP, but also forms acetyl CoA and NADPH for macromolecular synthesis.

Surgical resection followed by adjuvant chemotherapy is the only potentially curative treatment available, but resection is often difficult due to factors such as vascular involvement and boundary fuzziness^31,49^. The therapeutic treatment of PDAC is therefore challenging^1^. Progress has been unsatisfactory, and only a small increase in the overall survival has been achieved. This study also showed that the growth of pancreatic cancer could not be completely inhibited in an extreme hypoxic-ischemic environment. Hypoxia-induced heterogeneity of pancreatic cancer leads to the recurrence and invasion of these cells. Based on the spatial transcriptome characteristics of the hypoxia and control groups, we used CMAP to analyze the differences in chemosensitivities. The results showed that subgroup 6, the most invasive subgroup of the hypoxia group, was sensitive to ADZ-6482, a PI3K inhibitor^25^. The PI3K signaling pathway activates LDHA and promotes the occurrence of glycolysis. ATP is produced by glycolysis and the feedback mechanism of mitochondria resulting from PI3K-Akt-Foxo1 signaling. This promotes the continuous activation of the PI3K pathway and the further differentiation and proliferation of T cells^50,51^. It is suggested that the increased expression of LDHA in the most invasive subgroup of the hypoxia group activates the PI3K pathway, which promotes the invasion and metastasis of pancreatic cancer by regulating the differentiation, proliferation, metabolism, and stress of pancreatic cancer cells under hypoxic conditions (**Supplementary Figure S7**). PI3K inhibitors can inhibit the LDHA-induced activation of the PI3K signaling pathway^52,53^, which may become a new candidate drug for the treatment of pancreatic cancer. The efficacy of PI3K inhibitors in pancreatic cancer will be further verified in subsequent experiments. Therefore, an understanding of the heterogeneity in the microenvironment may highlight further the therapeutic strategies for PDAC.

In conclusion, this study is the first to characterize hypoxic microenvironment-induced changes in spatial heterogeneities in PDACs, and highlighted potential intercellular communication networks controlling the cell fate under different hypoxic conditions. In addition, the hypoxia gene signature and small chemical molecules identified by CMAP will serve as resources for further investigation of prognostic markers and tumor therapeutic targets.
