# Supplementary material for: Hypoxic microenvironment induced spatial transcriptome changes in pancreatic cancer
Source: Cancer Biol Med. 2021 Jun 15;18(2):616–30. doi: 10.20892/j.issn.2095-3941.2021.0158 (PMC8185871; doi:10.20892/j.issn.2095-3941.2021.0158)
Supplement: Supplementary file 1 [file cbm-18-616-s001.pdf]

Supplementary materials

Cells and reagents

Human pancreatic ductal adenocarcinoma (PDAC) panc-1 cells were cultured in RPMI-1640 medium with 10% fetal bovine serum, 4 mM L-glutamine, and 1% penicillin-streptomycin. Matrigel (BD Bioscience, San Jose, CA, USA) was diluted with RPMI-1640 medium for cell transplantation. The Hypoxyprobe-1 Kit (HP1-1000Kit) was purchased from HPI Hypoxyprobe (Burlington, VT, USA). Information of primary antibodies used in this study are listed in **Supplementary Table S1**. All secondary antibodies were provided by Zhongshan Golden Bridge Biotechnology (Beijing, China).

Tumor engrafted in the ischemic hind limbs of the nude mice model

The animal experiments were approved by the Tianjin Medical University Ethics Committee. All steps were carefully administered to protect the welfare of the animals and minimize suffering. Eight, 5-week-old female, NUDE mice were purchased from Beijing HFK Bioscience (Beijing, China). After 1 week of adaptation, the mice were randomly divided into 2 groups. Mice were anesthetized with 10% chloral hydrate. Then, the skin of the right groin was cut and the femoral artery and its branches were ligated, and the wound was sutured. The transplanted tumor was inoculated 24 h after surgery. Approximately 1–2 × 10^6, panc-1 cells were subcutaneously injected into the right groin of mice ($N = 4$). Tumors were measured every day, and the tumor volume was calculated using a standard formula (length × width^2 × 0.52). All mice were sacrificed when the average tumor volume reached 0.5 cm^3. Pimonidazole HCl was injected intraperitoneally (60 mg/kg) 30 min before the mice were sacrificed. The tumors and organs were then collected.

IHC staining

Paraffin-embedded sections were stained immunohistochemically. The sections were deparaffinized in xylene and rehydrated through graded alcohol solutions into water. Endogenous peroxidase was blocked with 3% hydrogen peroxide in 50% methanol for 10 min at room temperature. After rehydrating, the sections were washed with phosphate-buffered saline (PBS) and then pretreated with citrate buffer (0.01 M citric acid, pH 6.0) for 20 min at 100 °C. After rinsing with PBS, the slides were incubated with primary polyclonal antibodies, including the antibody against overnight at 4 °C (**Supplementary Table S1**). Visualization was performed using diaminobenzidine. Appropriate positive and negative controls were included.

Immunofluorescent staining

Serial frozen sections were used for immunofluorescent staining. Endogenous peroxidase was blocked using 5% goat serum at room temperature for 20 min. The slides were then incubated with primary anti-endomucin and anti-pimonidazole antibodies overnight at 4 °C, and then washed with PBS. An Alexa Fluor 680-labeled goat anti-rabbit secondary antibody (1:200, Invitrogen, Carlsbad, CA, USA) and a Texas red-labeled goat anti-rat secondary antibody (1:200, Invitrogen) were incubated with tissues at room temperature for 2 h, followed by washing with 2 washes of PBS. Stained tissue sections were mounted with a Vectashield mounting medium (ZLI.9557, Zhongshan, China) and were analyzed using a confocal microscope (A1; Nikon, Tokyo, Japan).

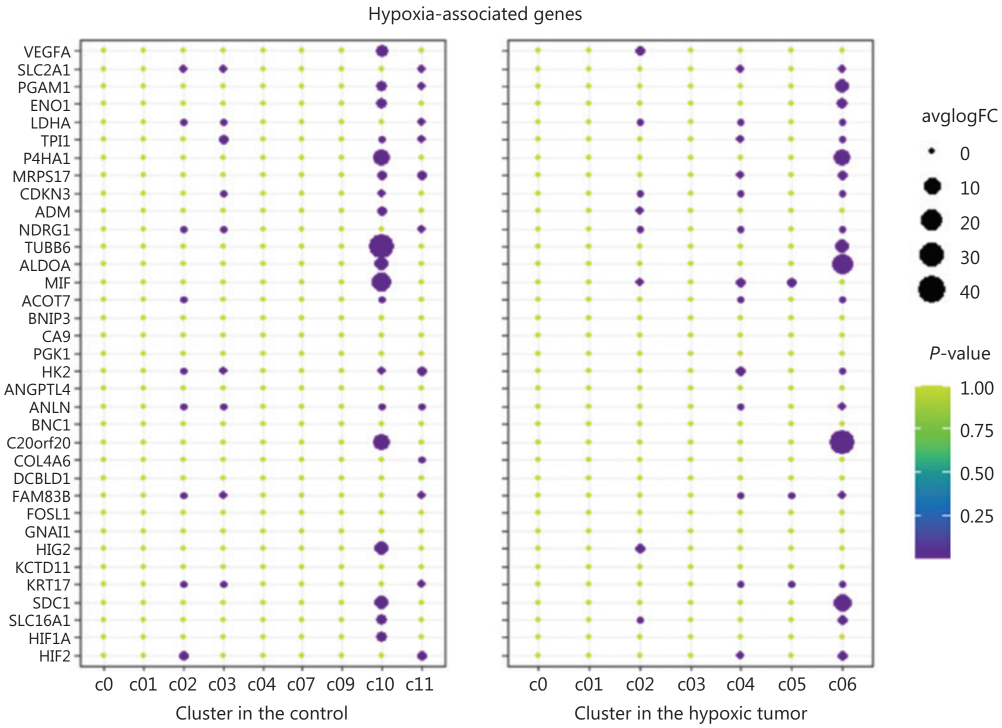

Figure S1 Distribution of hypoxia-related genes in different clusters of the hypoxia group.

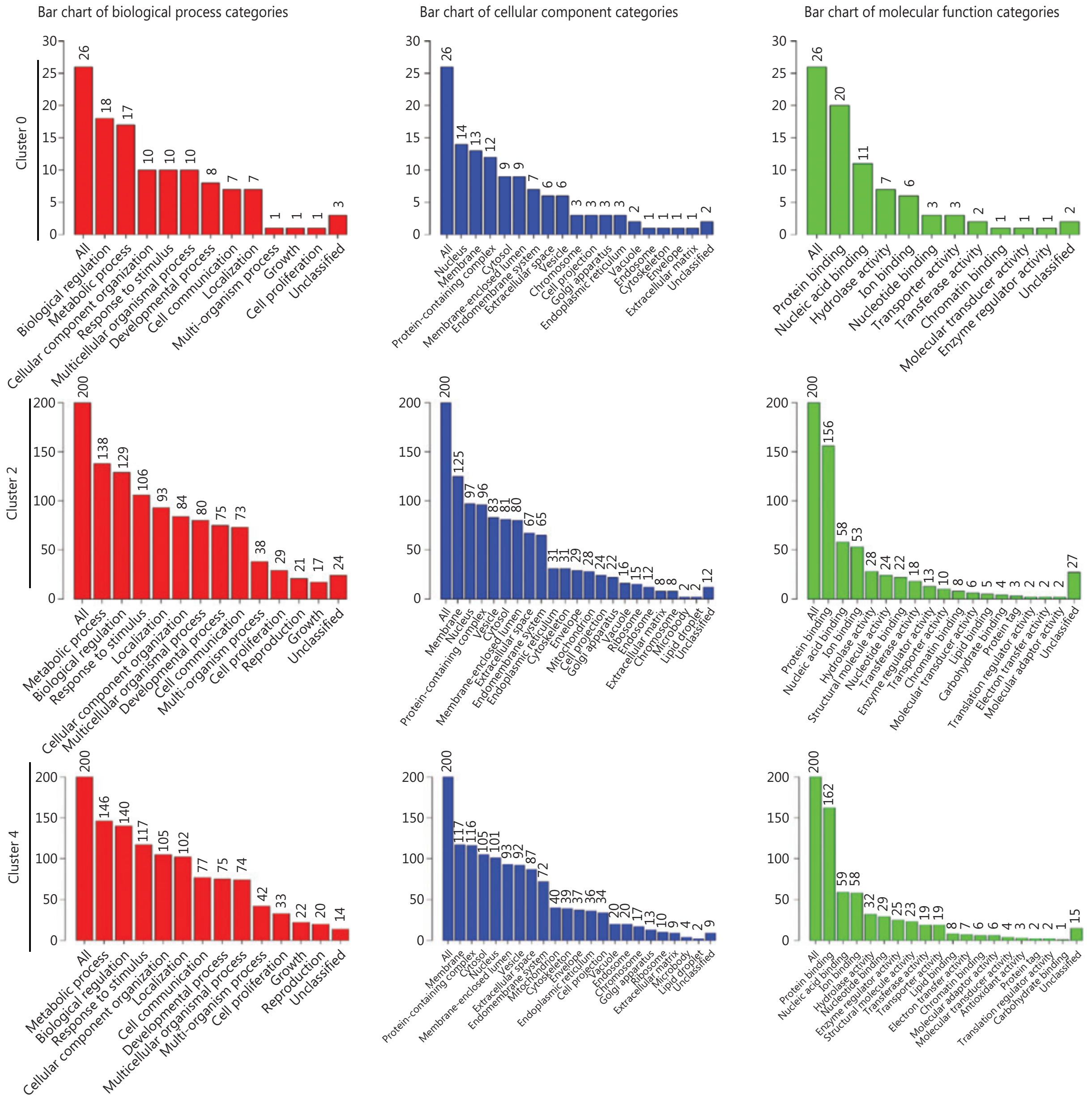

Figure S2 (continued)

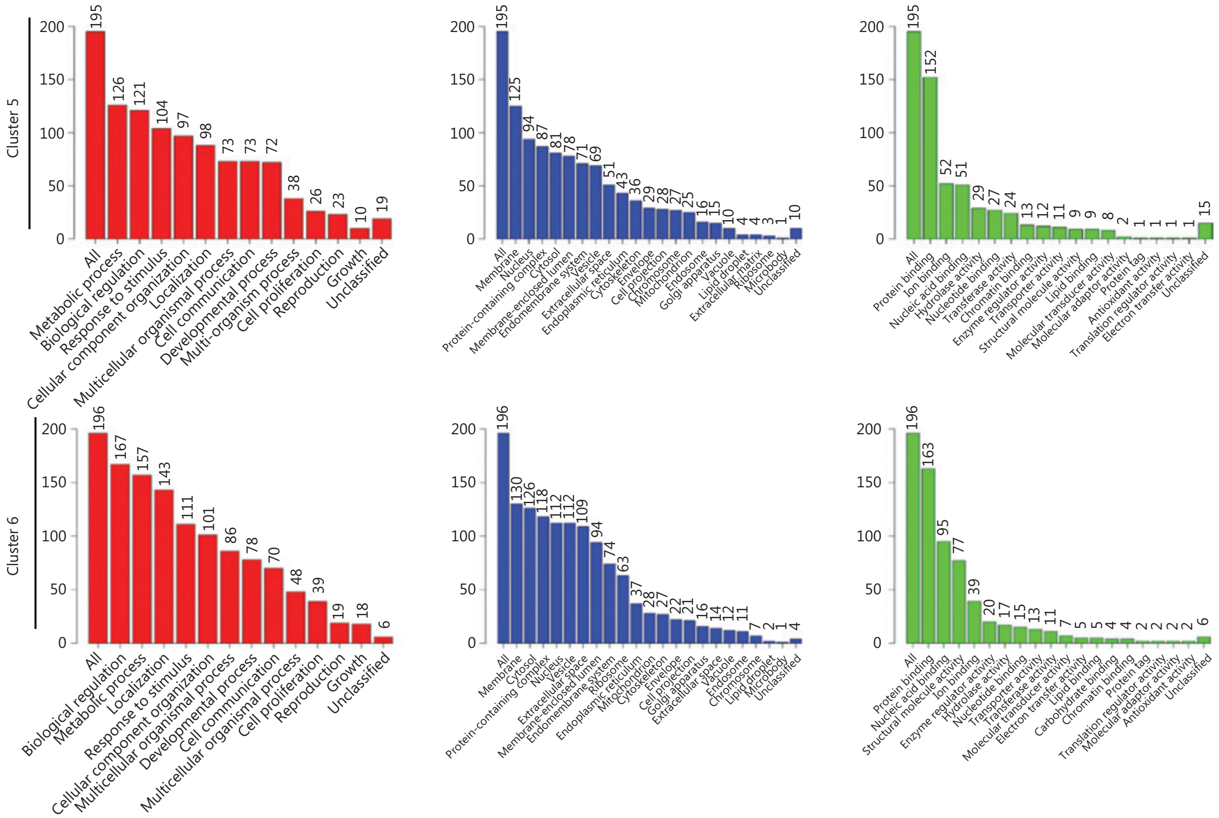

Figure S2 The characteristics of Kyoto Encyclopedia of Genes and Genomes enrichment pathway of clusters 0, 2, 4, 5, and 6 in the hypoxia group.

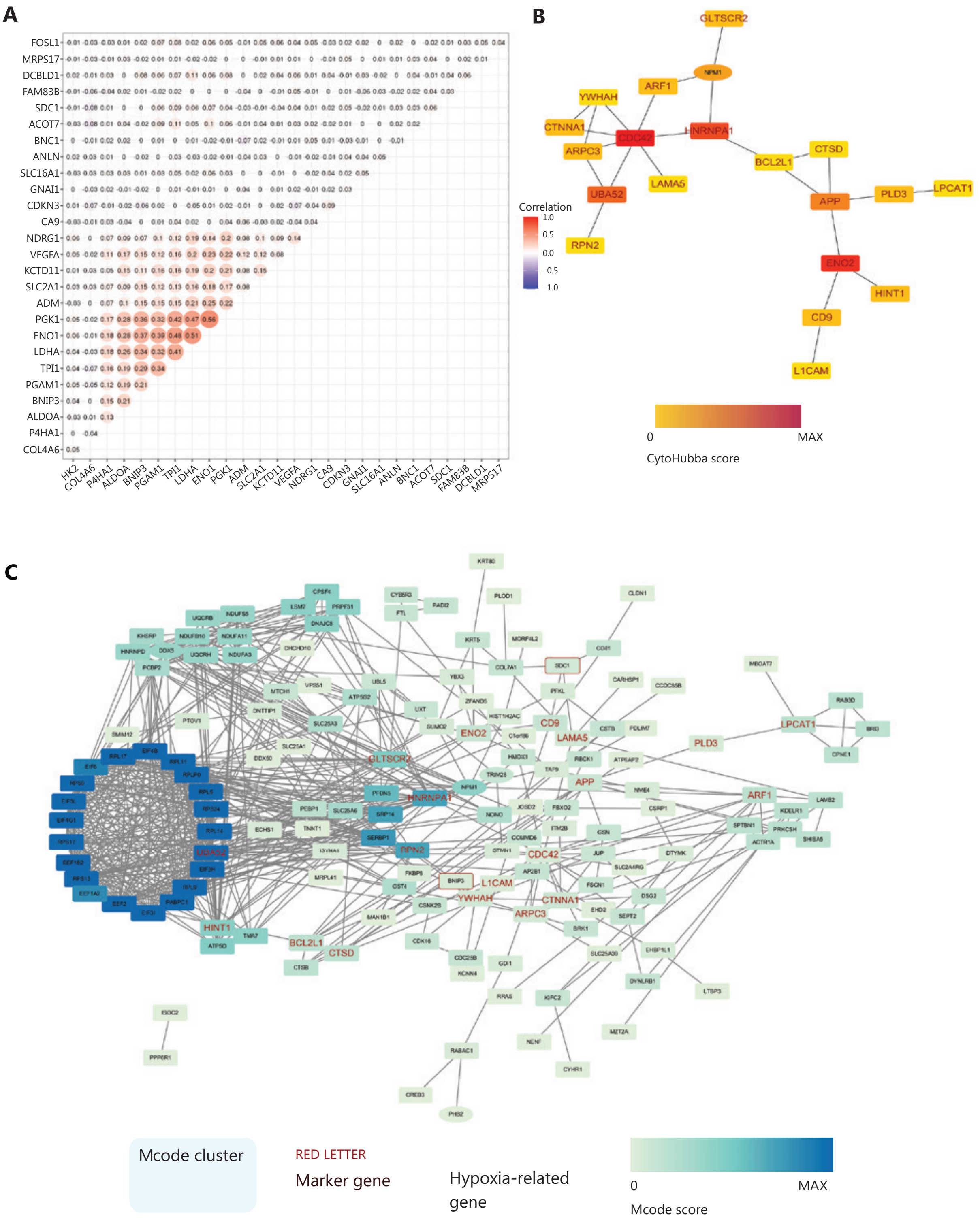

Figure S3 The co-essential relationship between differentially-expressed hypoxia genes and the top 100 genes in cluster 2 of the hypoxia group. (A) A correlation heat map of hypoxia genes in cluster 2 of the hypoxia group. (B) The network from the CytoHubba calculations in cluster 2 of the hypoxia group. (C) The co-essentiality network plot of differentially-expressed hypoxia genes and the top 100 genes in cluster 2 of the hypoxia group. The fill color of the node shows the ranking of Mcode scores. The cyana blue background indicates the Mcode group. The red indicates marker genes. The red border indicates differentially-expressed hypoxia genes.

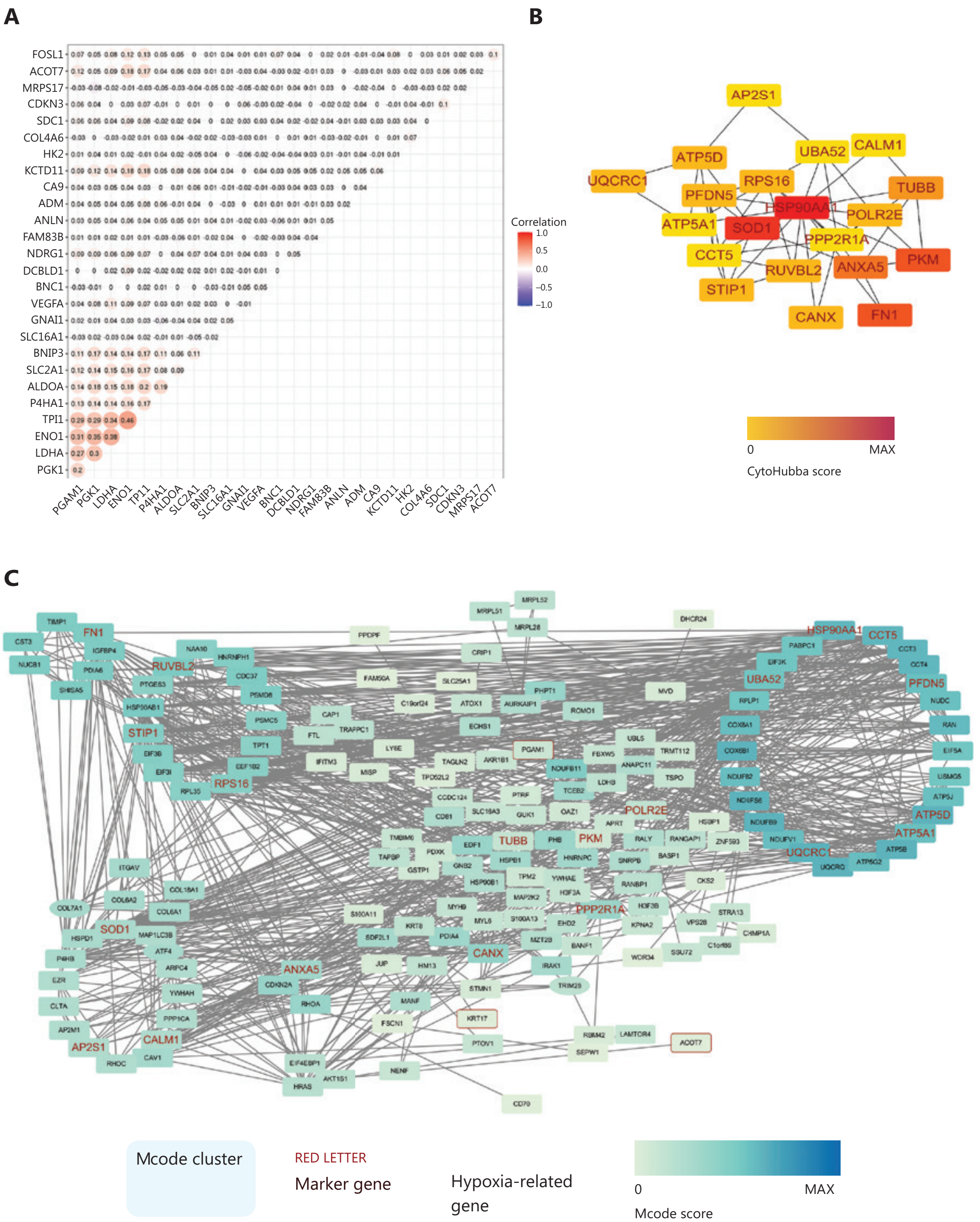

Figure S4 The co-essential relationship between differentially-expressed hypoxia genes and the top 100 genes in cluster 4 of the hypoxia group. (A) Correlation heat map of hypoxia genes in cluster 4 of the hypoxia group. (B) The network from the CytoHubba calculations for cluster 4 of the hypoxia group. (C) The co-essentiality network plot of differentially-expressed hypoxia genes and the top 100 genes in cluster 4 of the hypoxia group. The fill color of the node shows the ranking of the Mcode score. The cyana blue background indicates the Mcode group. The red indicates marker genes. The red border indicates differentially-expressed hypoxia genes.

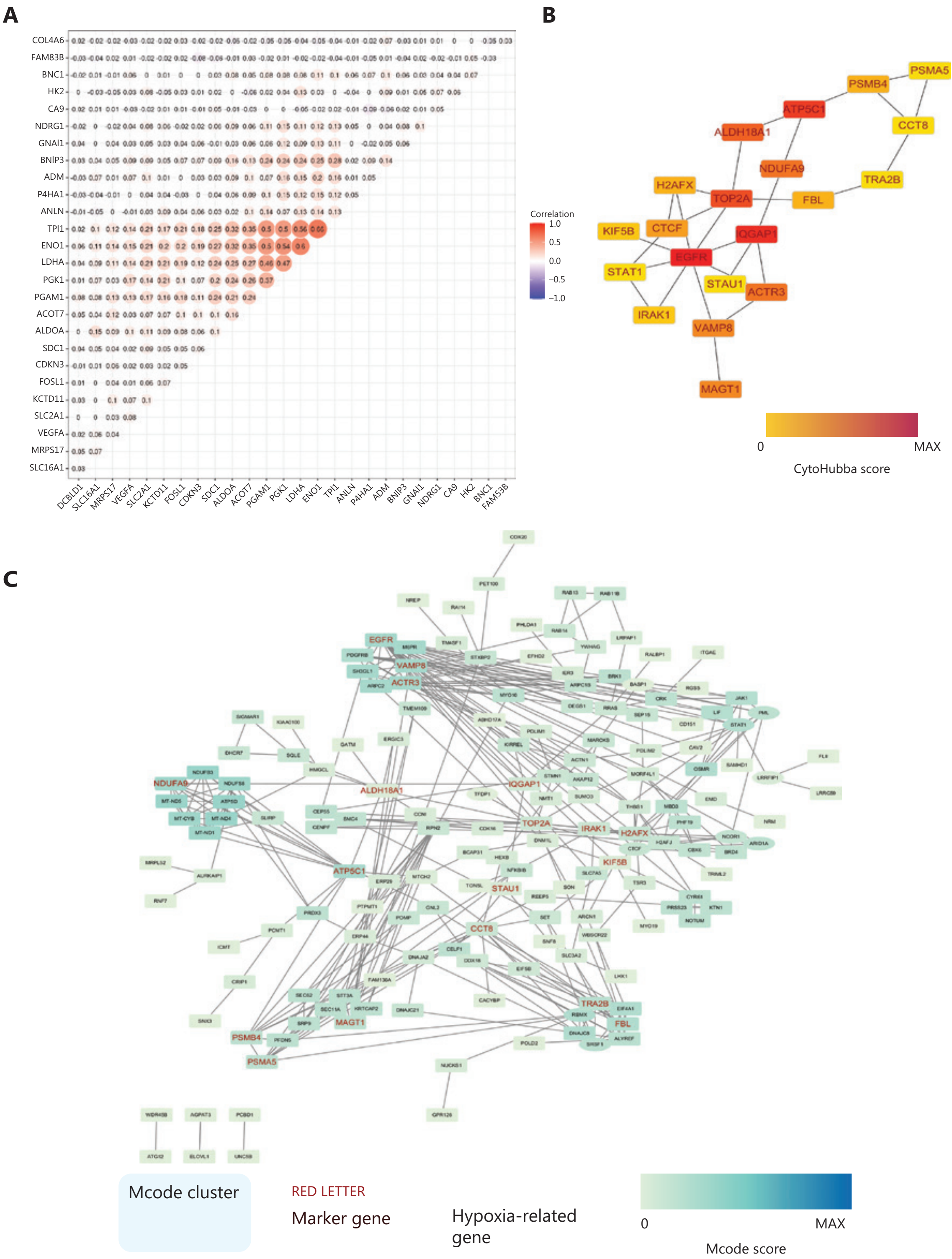

Figure S5 The co-essential relationship between differentially-expressed hypoxia genes and the top 100 genes in cluster 5 of the hypoxia group. (A) A correlation heat map of hypoxia genes in cluster 5 of the hypoxia group. (B) The network from the CytoHubba calculations in cluster 5 of the hypoxia group. (C) The co-essentiality network plot of differentially-expressed hypoxia genes and the top 100 genes in cluster 5 of the hypoxia group. The fill color of the node shows the ranking of the Mcode score. The cyana blue background indicates the Mcode group. The red indicates marker genes. The red border indicates differentially-expressed hypoxia genes.

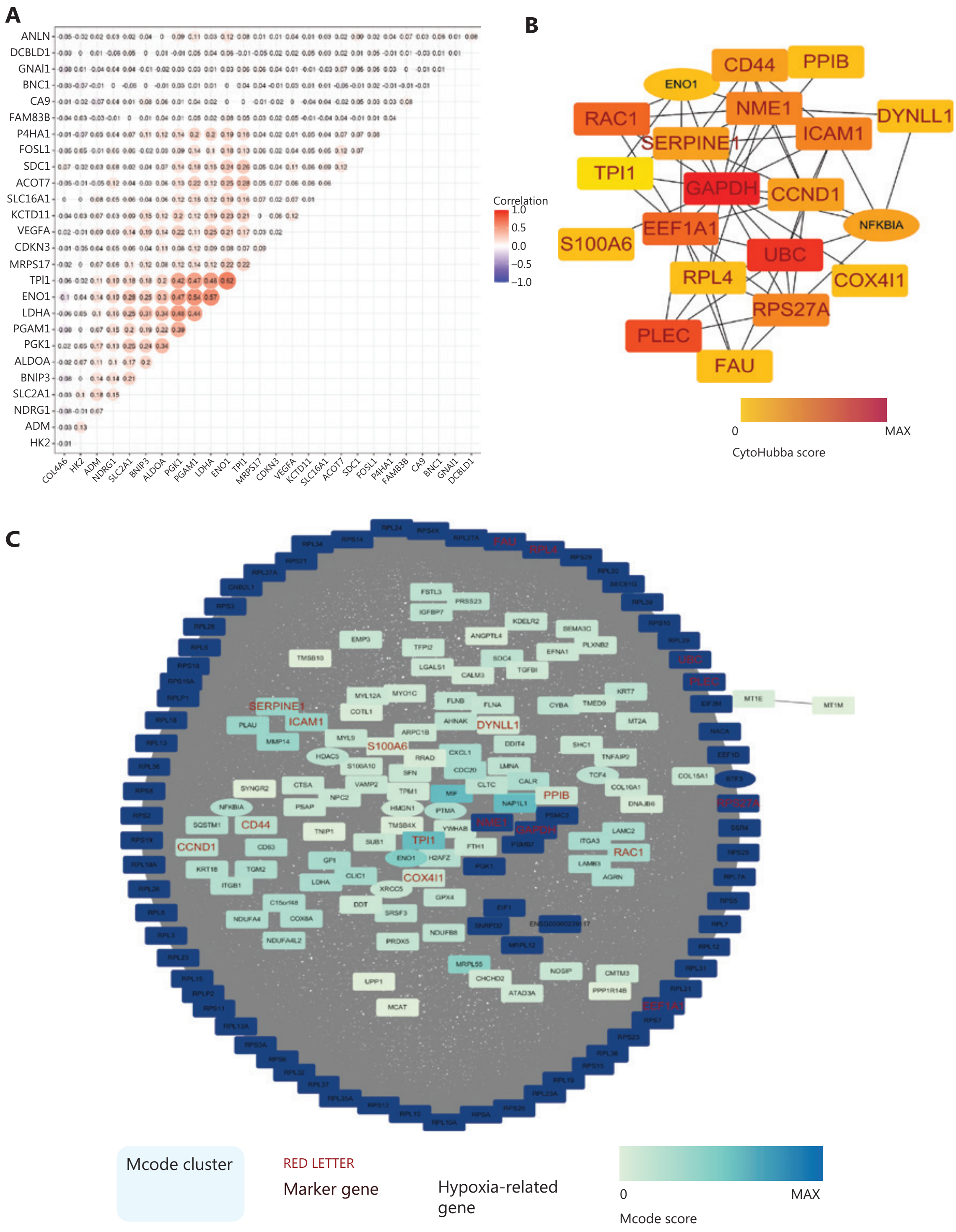

Figure S6 The co-essential relationship between differentially-expressed hypoxia genes and the top 100 genes in cluster 6 of the hypoxia group. (A) A correlation heat map of hypoxia genes in cluster 6 of the hypoxia group. (B) The network from the CytoHubba calculations in cluster 4 of the hypoxia group. (C) The co-essentiality network plot of differentially-expressed hypoxia genes and the top 100 genes in cluster 6 of the hypoxia group. The fill color of the node shows the ranking of the Mcode score. The cyana blue background indicates the Mcode group. The red letter indicates marker genes. The red border indicates differentially-expressed hypoxia genes.

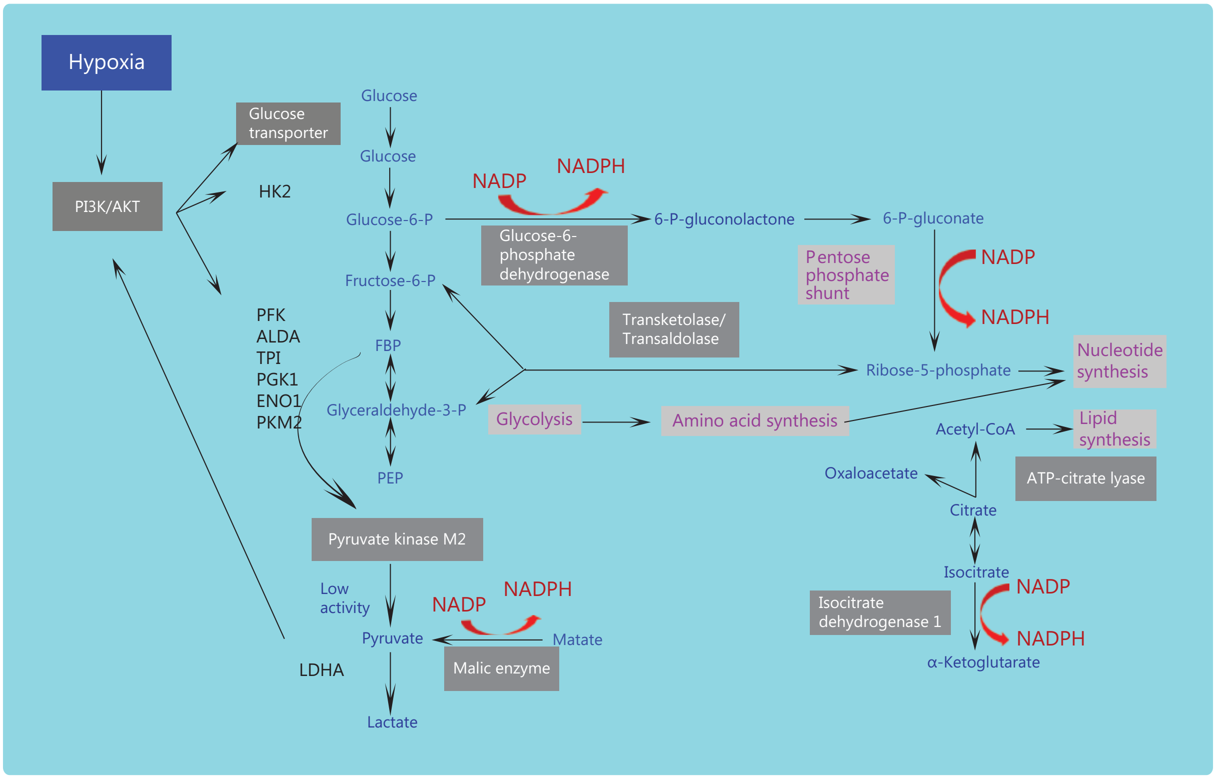

Figure S7 Graphical abstract. The hypoxia-induced PI3K signaling pathway activates LDHA and promotes glycolysis. ATP produced by glycolysis and mitochondria act on PI3K-Akt-Foxo1 in a feedback manner and promote the continuous activation of the PI3K pathway and further promote the invasion and metastasis of pancreatic cancer by regulating differentiation, proliferation, metabolism, and stress of pancreatic cancer cells under hypoxic conditions.

Table S1 The primary antibodies used in this study

Antibody	Source	NO.	Company	Dilution
LDHA	Rabbit	PA5-81292	Invitrogen	1:200
AKT	Rabbit	AF6259	Hinit	1:100
BNIP3	Rabbit	#44060S	CST	1:300
ALDOA	Rabbit	ab252953	Abcam	1:200
Endomucin	Rat	11-5851-80	eBioscience	1:400

Table S2 The hypoxia-related genes

Hgnc symbol	Names	Function
VEGFA	Vascular endothelial growth factor A	VEGF signalling
SLC2A1	Solute carrier family 2, member 1	Adipocytokine signalling
PGAM1	Phosphoglycerate mutase 1	Glucose metabolism
ENO1	Enolase 1	Glucose metabolism
LDHA	Lactate dehydrogenase A	Glucose metabolism
TPI1	Triosephosphate isomerase 1	Glucose metabolism
P4HA1	Prolyl 4-hydroxylase,α-polypeptide I	Extracellular matrix metabolism
MRPS17	Mitochondrial ribosomal protein S17	Mitochondrial translation
CDKN3	Cyclin-dependent kinase inhibitor 3	Cellular proliferation
ADM	Adrenomedullin	Signal transduction
NDRG1	N-myc downstream regulated 1	Response to metalion
TUBB6	Tubulin, β6	Gap junction
ALDOA	Aldolase A, fructose-bisphosphate	Glucose metabolism
MIF	Macrophage migration inhibitory factor	Tyrosine metabolism
ACOT7	Acyl-CoA thioesterase 7	Lipid metabolism
BNIP3	BCL2/adenovirus E1B 19 kDa protein-interacting protein 3	pro-apoptosis
CA9	carbonic anhydrase 9	pH regulation
PGK1	phosphoglycerate kinase 1	Glucose metabolism
HK2	Hexokinase2	Glycolysis, gluconeogenesis, energy pathway
ANGPTL4	angiopoietin-like 4	Lipid and glucose metabolism
ANLN	Anillin	Cytokinesis
BNC1	basonuclin 1	Keratinocyte proliferation
C20orf20	chromosome 20 open reading frame 20	Cellular proliferation
COL4A6	collagen, type IV, alpha 6	Extracellular matrix metabolism
DCBLD1	discoidin,CUB and LCCL domain containing 1	Unknown
FAM83B	family with sequence similarity 83, member B	EGFR signaling pathway; RAS/MAPK,PI3K/ AKT/TOR downstream signaling pathway
FOSL1	FOS-like antigen 1	Cellular proliferation
GNAI1	guanine nucleotide binding protein	Signal transduction
HIG2	hypoxia-inducible gene 2	Stress response
KCTD11	potassium channel tetramerization domain containing 11	Apoptosis
KRT17	keratin17	Keratin production
SDC1	syndecan 1	Cellular proliferation
SLC16A1	solute carrier family 16 member 1 (monocarboxylic acid transporter 1)	Glucose metabolism
HIF1A	hypoxia-inducible factor 1 alpha	Transcriptional regulator of the adaptive response to hypoxia
HIF2	EPAS1;Endothelial PAS Domain Protein 1	Regulating VEGF expression
